# Generation of a zebrafish SWATH-MS spectral library to quantify 10,000 proteins

**DOI:** 10.1038/sdata.2019.11

**Published:** 2019-02-12

**Authors:** Peter Blattmann, Vivienne Stutz, Giulia Lizzo, Joy Richard, Philipp Gut, Ruedi Aebersold

**Affiliations:** 1 Department of Biology, Institute of Molecular Systems Biology, ETH Zurich, Auguste-Piccard-Hof 1, 8093 Zurich, Switzerland; 2 Nestlé Institute of Health Sciences, EPFL Innovation Park, Bâtiment H, 1015 Lausanne, Switzerland; 3 Faculty of Science, University of Zurich, Zurich, Switzerland

**Keywords:** Proteomics, Mass spectrometry, Proteomic analysis, Zebrafish

## Abstract

Sequential window acquisition of all theoretical mass spectra (SWATH-MS) requires a spectral library to extract quantitative measurements from the mass spectrometry data acquired in data-independent acquisition mode (DIA). Large combined spectral libraries containing SWATH assays have been generated for humans and several other organisms, but so far no publicly available library exists for measuring the proteome of zebrafish, a rapidly emerging model system in biomedical research. Here, we present a large zebrafish SWATH spectral library to measure the abundance of 104,185 proteotypic peptides from 10,405 proteins. The library includes proteins expressed in 9 different zebrafish tissues (brain, eye, heart, intestine, liver, muscle, ovary, spleen, and testis) and provides an important new resource to quantify 40% of the protein-coding zebrafish genes. We employ this resource to quantify the proteome across brain, muscle, and liver and characterize divergent expression levels of paralogous proteins in different tissues. Data are available via ProteomeXchange (PXD010876, PXD010869) and SWATHAtlas (PASS01237).

## Background & Summary

Proteins execute most cellular processes and thus define the phenotype of cells and tissues^1^. Whereas transcript abundance can be used to infer cellular activities to some extent, proteomic data generally explains differences in phenotypes more accurately^2–4^. SWATH-MS is a mass spectrometry method that can be employed to reproducibly quantify the proteome across a large number of biological samples as it combines data-independent acquisition (DIA) with a peptide-centric data query strategy^5–8^. This proteomic method has been systematically benchmarked and has shown to produce highly reproducible results when measuring the same samples in various laboratories and when analyzing the same data with various software tools^9,10^. SWATH-MS thus represents an ideal proteomic method for large-scale and reproducible quantification of the proteome across many biological samples that can be used to understand the molecular mechanisms defining complex physiological phenotypes.

Importantly, SWATH-MS requires a spectral library containing SWATH assay coordinates to specifically extract the peptide quantities from the multiplexed mass spectrometry data^5,11,12^. Alternative approaches such as DIA-Umpire or PECAN exist to query mass spectrometry data acquired in data-independent acquisition (DIA) mode without the need of a spectral library, but until now they have proven less sensitive^10,13,14^. Whereas a study-specific SWATH spectral library can be generated with moderate effort, using large previously assembled spectral libraries that are shared by the community has, among other things, the advantage of reducing the amount of sample and measurement time typically by 50% and of supporting protein identifications with a consistent set of reference spectra. To efficiently control the false discovery rate (FDR) when using such large spectral libraries, various post-analysis approaches have been developed^15,16^. Large SWATH spectral libraries containing coordinates to quantify over 5,000 proteins have been generated and publicly deposited for organisms such as humans and drosophila^17,18^, but for zebrafish no large SWATH spectral library exists yet.

Zebrafish is a rapidly emerging vertrebrate model system used in many fields of biology and physiology^19^. In contrast to other model organisms such as mice, zebrafish are not isogenic and the commonly used lines contain a genetic diversity estimated to be similar to that in the human population^20^. Hence, zebrafish is a particular interesting model organism to assess inter-individual variability and a comprehensive SWATH spectral library would efficiently support such studies by allowing the accurate measurement of the proteome across zebrafish tissues of individual fish. The zebrafish genome encodes about 25,500 protein-coding genes^21^. Less than 5% of tryptic peptides are shared despite many zebrafish genes being homologous to human genes. In total, 58% of the human protein-coding genes have one zebrafish orthologue; an additional 15% of human protein-coding genes have two or more orthologuos genes in zebrafish. The high number of genes with two orthologs is due to a whole-genome duplication that occurred in the teleost ancestors of zebrafish^22^. These duplicated genes, also called ohnologues, subsequently evolved during ~320–350 mio years independently and represent interesting opportunities to learn more about evolution and acquired protein functions^23^.

**Figure 1:**
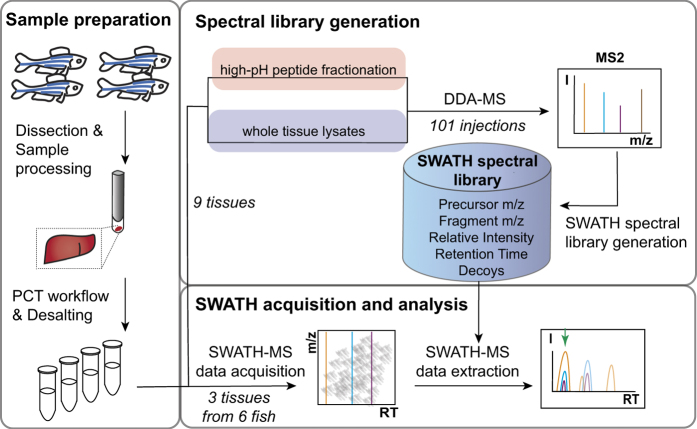
Workflow of creating and using the SWATH spectral library. Samples were prepared using the pressure-cycling (PCT) workflow^24^. The spectral library was built from fragment ion spectra generated by data-dependent acquisition mass spectrometry (DDA-MS) from fractionated and unfractionated peptide samples^11^. The spectral library was then used to analyze samples from 3 different tissues using the OpenSWATH workflow^12^.

Here we present a large SWATH spectral library for zebrafish with coordinates to quantify 10,405 proteins and thus 40.4% of the predicted protein-coding zebrafish genes. The library was generated by combining the results from 101 injections of 83 peptide samples obtained from both fractionated and unfractionated peptide mixtures extracted from 9 different zebrafish tissues (Fig. 1 and Table 1). These samples were processed using the pressure-cycling technology (PCT) that allowed the reproducible lysis and digestion of minute amounts of tissue^24^. The spectral library is deposited on ProteomeXChange (Data Citations 1, 2) and SWATHAtlas (Data Citation 3). We demonstrate the utility of the SWATH spectral library by analyzing the zebrafish proteome in three different tissues and characterize the tissue-specific protein expression of several ohnologues.

## Methods

### Zebrafish husbandry and tissue dissection

Adult AB zebrafish were raised at 28 °C under standard husbandry conditions. All experimental procedures were carried out according to the Swiss and EU ethical guidelines and were approved by the animal experimentation ethical committee of Canton of Vaud (permit VD3177). The 6 month old male and female zebrafish were euthanized, manually dissected, and tissues were snap-frozen in liquid nitrogen for further processing following standard protocols^25^.

### Sample preparation

Brain, eye, testis, and ovary from male or female zebrafish, and the muscle from male zebrafish were cut into sections or grinded while cooling with liquid nitrogen. From the grinded tissues, about 3 mg were transferred into pressure cycling technology (PCT) Microtubes (Pressure BioSciences). For spleen, liver, heart and intestine of male or female zebrafish, a piece of 0.9–3.8 mg of tissue was transferred into PCT Microtubes for subsequent processing. For all samples, lysis and digestion were performed based on a protocol described in Guo *et al.*^24^. Lysis buffer, pH 8.0 (6 M urea, 2 M thiourea, 100 mM ammonium bicarbonate, 5 mM EDTA, cOmplete™ protease inhibitor (1:50)) was added to the tissue sections, and lysis and digestion of the samples was performed with the Barocycler NEP2320EXT (Pressure BioSciences) at 31 °C. Lysis was conducted using the PCT-MicroPestle with 60 cycles consisting of 50 s at 45 kpsi followed by 10 s at atmospheric pressure. For reduction and alkylation, the buffer was diluted to 3.75 M urea with 100 mM ammonium bicarbonate, before peptides were simultaneously reduced with 9 mM tris(2-carboxyethyl)phosphine (TCEP) and alkylated with 35 mM iodoacetamide for 30 min in the dark at 25 °C. The first digestion step was performed using LysC (estimated enzyme/protein ratio of 1:100; Wako Chemicals), and was carried out in the barocycler using 45 cycles of 50 s at 20 kpsi followed by 10 s at atmospheric pressure. For the second digestion, samples were diluted to 2 M Urea with 100 mM ammonium bicarbonate and digested using Trypsin (estimated enzyme/protein ratio of 1:75; Promega) for 90 cycles consisting of 50 s at 20 kpsi followed by 10 s at atmospheric pressure. The digestion was quenched by acidifying samples to pH 1.5 with trifluoroacetic acid (TFA). The peptides were desalted using C18-columns (The Nest Group Inc.) and 2% (v/v) acetonitrile and 0.1% (v/v) TFA in water and eluted with 50% (v/v) acetonitrile and 0.1% (v/v) TFA in water. The buffer was evaporated using vacuum centrifugation at 45 °C. Dried peptides were either dissolved in 2% (v/v) acetonitrile and 0.1% (v/v) formic acid (FA) in water supplemented with iRT peptides (Biognosys, Schlieren) for injection into the mass spectrometer, or they were prepared for high-pH RP-HPLC fractionation.

### High-pH fractionation of peptides

Samples for high-pH RP-HPLC fractionation were resuspended in Buffer A (20 mM ammoniumformate and 0.1% ammonia solution in water, pH 10) and 80 μg of peptides were injected into an Agilent Infinity 1260 (HP Degasser, Vial Sampler, Cap Pump) and 1290 (Thermostat, FC-μS) system. The peptides were separated at 30°C on an YMC-Triart C18 Reversed Phase Column with diameter of 0.5 mm, length of 250 mm, particle size of 3 μm, and pore size of 12 nm. At a flow of 11 μL/min the peptides were separated by a linear 56 min gradient from 5% to 35% Buffer B (20 mM ammoniumformate, 0.1% ammonia solution, 90% acetonitrile in water, pH 10) against Buffer A followed by a linear 4 min gradient from 35% to 90% Buffer B against Buffer A and 6 min at 90% Buffer B. The resulting 36 fractions per organ were pooled based on the collection order from fraction 3 to fraction 33 or 34 (depending on the UV profile) into 8 samples by the following scheme: fraction x was pooled with fractions x + 8, x + 16, and x + 24. The buffer of the pooled samples was evaporated using vacuum centrifugation at 45 °C. The peptides were dissolved in 2% (v/v) acetonitrile and 0.1% (v/v) FA in water supplemented with iRT peptides (Biognosys, Schlieren) for injection into the mass spectrometer.

### DDA acquisition of samples

The peptides were quantified on an ABSciex TripleTOF 5600 instrument after separating 0.9–3 μg by nano-flow liquid chromatography (NanoLC Ultra 2D, Eksigent). The peptides were separated by reverse-phase chromatography on a fused silica PicoTip™ Emitter (inner diameter 75 μm) (New Objective, Woburn, USA) manually packed column with C18 beads (MAGIC, 3 μm, 200 Å, BISCHOFF, Leonberg, Germany) to a length of 20 cm for the whole lysate samples, and 30 cm for the pooled fractions. A flow of 300 nl/min and a linear 120 min gradient from 2% to 35% Buffer B (98% acetonitrile and 0.1% formic acid in H_2_O) in Buffer A (2% acetonitrile and 0.1% formic acid in H_2_O) was used to separate the peptides. Precursor selection on the MS1 level was performed with a Top20 method using an accumulation time of 250 ms and a dynamic exclusion time of 20 s. The MS1 spectra were obtained in an m/z range from 360 to 1460. Fragmentation of the precursor peptides was achieved by collision induced dissociation (CID) with rolling collision energy for peptides with charge 2+ adding a spread of 15 eV. For MS2 spectra, only fragments with a charge state from 2 to 5 were selected using an accumulation time of 150 ms.

### Building the SWATH spectral library

The SWATH spectral library was built using the previously published workflow^11^ with some modifications of the search engines, number of missed cleavages, mass errors, and selection of proteins and peptides by the iProphet cutoffs. First, raw files were converted into centroided mzXML files with ProteoWizard version 3.0.8851. The spectra were then searched using an in-house pipeline employing the search engines X!Tandem with k-score plugin (2013.06.15.1) and Comet (2016.01 rev.3) against a protein sequence database. The protein sequence database was obtained from the Ensembl Release 91 (dec2017.archive.ensembl.org; Danio_rerio.GRCz10.pep.all.fa) and further processed using an R script to select only the longest protein-coding transcript for each protein-coding gene. The search was conducted on an in-house platform^26^ using as search parameters a parent mass error of ±25 ppm, a fragment mass error of ±0.05 Da, trypsin digestion allowing for 2 missed cleavages, carbamidomethyl (C) as a fixed modification, and oxidation (M) as a variable modification. After combining the searches, only proteins passing an iProphet probability corresponding to a Mayu^27^ protein-FDR of 0.010 were selected. For these proteins, all peptides passing an iProphet peptide-FDR<0.0100 were selected using SpectraST (v.5.0). A consensus spectral library was generated with retention time normalization using iRT peptides and this spectral library was then used to generate the SWATH spectral library^11^.

### Quantitative analysis of tissue samples

The peptides of brain, liver, and muscle of 6 male wild type zebrafish (6 months old) were quantified as described above for the fractionated samples with the difference that a 90 min gradient was used and the mass spectrometer was operated in SWATH mode. The precursor peptide ions were accumulated for 250 ms in MS1 and fragmented in 64 overlapping variable windows within an m/z range from 400 to 1200. Fragmentation of the precursor peptides was achieved by Collision Induced Dissociation (CID) with rolling collision energy for peptides with charge 2+ adding a spread of 15 eV. The MS2 spectra were acquired in high-sensitivity mode with an accumulation time of 50 ms per isolation window resulting in a cycle time of 3.5 s. The tissues from the different fish were injected consecutively in a block design to prevent any possible confounding effects due to deviation in machine performance. The SWATH-MS data was quantified using the OpenSWATH workflow^12^ on the in-house iPortal platform^26^. An m/z fragment ion extraction window of 0.05 Th, an extraction window of 600 s, and a set of 10 different scores were used as described before^12^. To match features between runs, detected features were aligned using a spline regression with a target assay FDR of 0.01^28^. The aligned peaks were allowed to be within 3 standard deviations or 60 s after retention time alignment. For runs where no fragment ion peaks for a specific query peptide could be identified, the signal was requantified and was assigned an m-score of 2^28^. The data was then further processed using the R/Bioconductor package SWATH2stats^15^. Proteins that had precursors with an m-score lower than 1.4125 × 10^−8^ and peptides with an m-score threshold lower than 7.0795 × 10^−6^ were selected for further analysis. This threshold resulted in an estimated peptide FDR of 0.00991, and protein FDR of 0.0092 (using an estimated fraction of false targets (FFT) or π_0_-value of 0.765 for estimating the FDR). In total 29,916 peptides passed this stringent threshold. The protein abundance was then estimated using the IBAQ method with the aLFQ R/CRAN package^29^.

### Code availability

The code necessary to build the SWATH spectral library has been described in detail in a recent publication^11^. The workflows to analyze SWATH-MS data have been published^12,15,28^ and are described on http://www.openswath.org.

## Data Records

The raw mass spectrometry DDA files for library generation, the search results (pepXML), the consensus spectral library, and the SWATH spectral library have been deposited to the ProteomeXchange Consortium via the PRIDE partner repository^30^ (Data Citation 1). In addition, the zebrafish SWATH spectral library is available through the SWATHAtlas repository in different formats and with different precursor window settings (Data Citation 3).

The raw mass spectrometry DIA files for quantifying the proteome across muscle, liver, and brain, the data matrix obtained from the OpenSWATH analysis and the results from the aLFQ/IBAQ estimation have been deposited to the ProteomeXchange Consortium via the PRIDE partner repository^30^ (Data Citation 2).

## Technical Validation

### Controlling the false discovery rate

**Figure 2:**
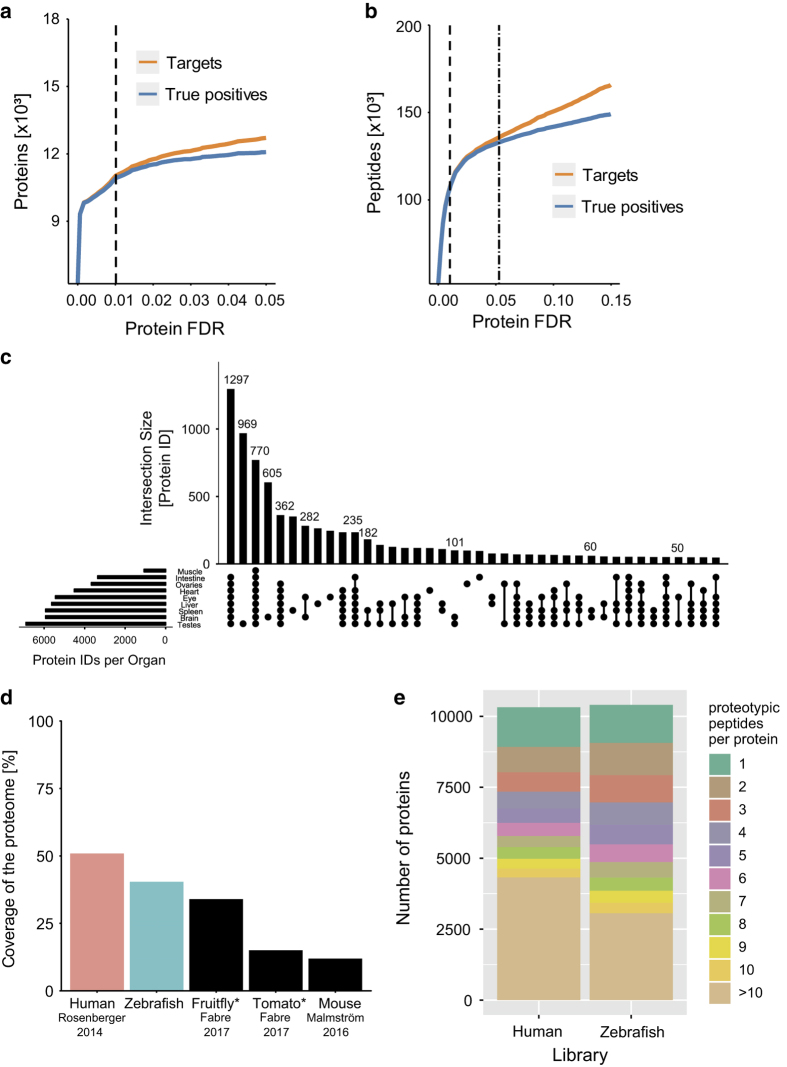
Characteristics of the zebrafish SWATH spectral library. (**a**) Number of true positive (blue) and target (orange) protein identifications at a given protein FDR. The applied MAYU protein-FDR cutoff is shown with a vertical dashed line. (**b**) Number of true positive (blue) and target (orange) peptide identifications at a given protein FDR. The applied MAYU protein FDR-cutoff of 0.01 is shown with a vertical dashed line. The dotted-dashed line indicates the peptide FDR threshold (0.01) that was applied to all peptides of proteins passing the protein cutoff. (**c**) Contribution of the individual organs to the number of identified proteins by proteotypic peptides. (**d**) Coverage of the proteome for SWATH spectral libraries of different species^17,18,39^. The coverage was calculated using the number of protein identifications with proteotypic peptides against the total number of proteins present in the sequence database, or the numbers from the cited publication were used (marked with an asterisk). (**e**) Barplot of the proteotypic peptides per protein in the human^17^ and the zebrafish library.

The false discovery rate (FDR) needs to be stringently controlled when performing bottom-up proteomics across many samples, because the false-positive identifications accumulate faster at protein-level compared to precursor-level^27^. We therefore employed the MAYU strategy^27^ to filter the protein list of our spectral library to a protein-FDR of 1% (Fig. 2a), as was done in other large SWATH spectral libraries^17^. For the proteins that passed this threshold, we then selected all the peptides that passed a more lenient iProphet^31^ probability cutoff that corresponded to a peptide FDR of 1%. The assay saturation curves (Fig. 2b) show that both thresholds are very stringent and the number of true assays did not reach saturation yet. Nevertheless, we maintained the stringent community standard to keep the false discovery rate at a minimal level.

### Protein sequence database

A key consideration when searching mass spectra is the selection of the protein sequence database. The UniprotKB/Swiss-Prot database has the advantage of providing stable and non-redundant protein identifiers^32^. However, as the UniprotKB/Swiss-Prot database for zebrafish currently contains only about 3000 reviewed entries, we opted to map our identified spectra to the Ensembl sequence database^21^. In contrast to the UniprotKB/Swiss-Prot database, Ensembl is a gene-centric database. To minimize the redundancy of the sequences from different protein identifiers, we selected for each of the 25,903 genes the peptide sequence corresponding to the longest protein-coding transcript. This resulted in a protein database containing 25,728 protein sequences that was used to search the mass spectra. The resulting spectral library contains assays for 104,185 proteotypic peptides from 10,405 proteins (Table 2). With these assays, 40.4% of the protein-coding genes of zebrafish can be quantified (Fig. 2d). Counting also the peptides shared by several proteins would increase the number of peptides by an additional 10,727 peptides or 10.3% (Table 2). In our subsequent analysis, only proteins quantified by proteotypic peptides were counted and no protein grouping using the Occam’s razor approach was performed. However, the assays for shared peptides are present in the library and can be analyzed if necessary. The number of proteins supported by proteotypic peptides is slightly larger in the zebrafish library than the combined assay library for humans^17^ while our library contains 30% fewer proteotypic peptides (Fig. 2e). A likely reason for the lower number of proteotypic peptides per protein is that we compiled our library from a three times lower number of mass spectrometry injections and that the human library included samples from affinity purifications that achieve a higher sequence coverage. Furthermore, the relative amount of shared peptides is nearly twice as high in the zebrafish SWATH spectral library (9.4%) than in the human SWATH spectral library (4.9%) and suggests that the genome duplication makes it more difficult to identify proteotypic peptides in zebrafish due to the ohnologues with highly similar peptide sequence. Based on the number of proteins it contains, this SWATH spectral library is currently the largest publicly deposited library on the SWATH Atlas repository and despite zebrafish being the vertebrate with the highest number of protein-coding genes, we achieve a good coverage of 40% of all protein-coding genes.

### SWATH spectral library from nine different tissues

Nine different tissues from zebrafish were processed to generate this library. Samples from brain, eye, heart, intestine, liver, muscle, ovary, spleen and testis were processed using the PCT workflow^24^ and acquired in data-dependent acquisition mode on a TripleTOF 5600 instrument. The organ contributing most identifications was testis. In total, 969 (9%) of the proteins in the library were exclusively detected in testis (Fig. 2c). In contrast, muscle tissue contributed the lowest number of identifications reflecting the challenge of proteomic measurements in a tissue in which few highly abundant proteins make up most of the protein mass^33^. Nevertheless, we have recently shown the potential of measuring the proteome in such challenging tissues by characterizing exercise-induced changes in zebrafish muscle^34^. We thus envision the zebrafish SWATH spectral library to support and facilitate SWATH-MS studies in various tissues of this emerging model organism.

### Reproducibility of coordinates in SWATH spectral library

**Figure 3:**
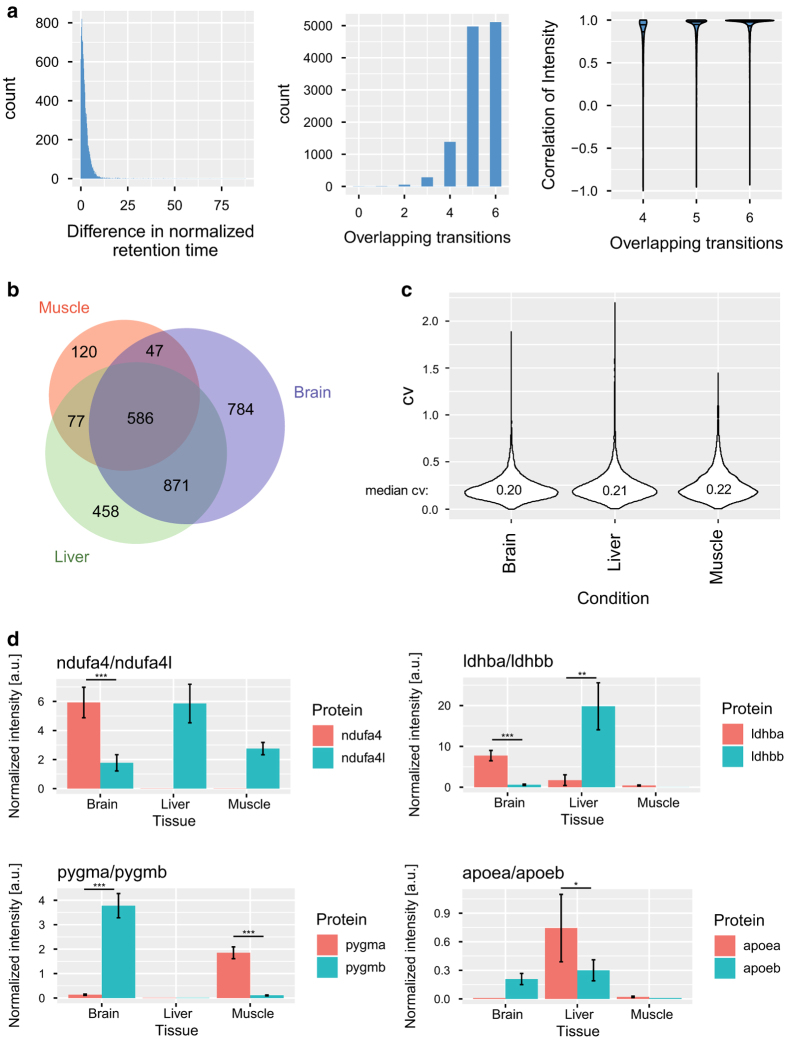
Quantification of the zebrafish proteome across brain, muscle and liver. (**a**) Comparison of the SWATH assay coordinates to quantify the 10,115 peptides common to both the zebrafish and human SWATH spectral library^17^. Plotted are the difference in retention time normalized to iRT peptides^37^ for the 11,816 precursor ions present in both libraries, how many of the 6 fragment ions per precursor (70,896 fragment ions in total) are identical, and the correlation of the relative intensities for the 11,463 precursor ions with at least 4 overlapping transitions. The horizontal lines in the violin plots depict the quartiles. (**b**) Overlap of proteins quantified across three different zebrafish tissues. (**c**) Coefficient of variation of the quantified signal for protein abundance across 6 fish. (**d**) Protein abundance of ohnologues across the three zebrafish tissues. The error bars represent standard deviation of six different fish and the difference in abundance was tested using an unpaired t-test for ohnologues quantified in the same tissue (^∗∗∗^adj. p-value<0.001, ^∗∗^adj. p-value<0.01, ^∗^adj. p-value<0.05, n.s. adj. p-value>0.05).

To assess the similarity of the coordinates for the peptides contained in the SWATH spectral library, we compared the coordinates for the peptides present in both our library and the human SWATH spectral library^17^. In total, 11,816 precursor ion signals from 10,115 peptides (10%) were present in both libraries (Fig. 3a). For 85% of these, at least 5 of the 6 selected transitions were identical. For 90% of the precursor ion signals, the difference in retention time normalized to the iRT peptides was less than 5, corresponding to a difference in retention time of about 3.2 min on a 90 min gradient. Moreover, the median Pearson’s correlation between the intensity of the transition signals of shared peptides was 0.98 for the precursors and generally increased as a higher number of transitions were shared (Fig. 3a). These results demonstrate that the coordinates that are used to measure the peptide abundance are very similar in the two SWATH spectral libraries, even though they were obtained from very different samples and at different times.

### Quantification of proteins across tissues

To show the utility of the zebrafish SWATH spectral library, we compared the proteomes of muscle, brain and liver from wild-type zebrafish with respect to the composition and quantity of proteins. Samples from six different fish were dissected and processed using our developed PCT workflow and analyzed in SWATH-MS mode. The quantitative data were extracted with OpenSWATH^12^ using the SWATH spectral library described above. More than 2,900 proteins were quantified passing our stringent filters of which 1,581 (54%) proteins were quantified in at least two tissues (Fig. 3b). The median coefficient of variation for the intensity of the 36,247 quantified peak groups across the 6 different zebrafish was 20.9% (Fig. 3c). This shows that the spectral library can be used to efficiently and reproducibly quantify thousands of proteins across different tissues using SWATH-MS. All the protein quantities and statistical comparisons between different tissues have been deposited as a data resource for further analysis (Data Citation 2). For this manuscript, we choose to highlight the potential of such an analysis at the example of ohnologues.

### Divergent expression of ohnologues

Ohnologues are paralogues genes and proteins that have appeared after a whole genome duplication event. The ancestor of zebrafish underwent such a whole genome duplication, termed the teleost specific genome duplication (TSD)^22^. As a result, more than 2500 human protein-coding genes have two orthologs in zebrafish. From 2659 pairs of ohnologues, we quantified both in at least one tissue for 160 instances. For 25 (16%) of these, we find an at least 2-fold difference in expression between the two ohnologues. The dominant ohnologue varies across the different tissues suggesting that the two paralogues may have acquired tissue-specific functions leading to the evolution of this observed difference in regulation (Fig. 3c). For example, ndufa4 is expressed in brain at a 3-fold higher level than ndufa4l, whereas ndufa4l is the dominant protein version expressed in liver and muscle. Ndufa4 is a member of the electron transport chain in mitochondria and we recently described that it interacts with respiratory supercomplexes in zebrafish^34^. The amino acids of ndufa4 and nduf4l are 73% and 68% conserved to the human NDUFA4 orthologue, but 24% of the amino acids differ among the two paralogues. It is not clear if these ohnologues possess different activity or functionality, but the different expression levels could suggest that the duplicated proteins acquired divergent roles in the different tissues.

## Usage Notes

### Sample preparation

Our samples were processed using the pressure-cycling technology (PCT) that allowed the reproducible lysis and digestion of minute amounts of tissue^24^. However, our spectral library is compatible with any other lysis and digestion protocol as long as a complete lysis, reduction and alkylation of cysteine bonds, and digestion is ensured. When processing very small amounts of tissue, special care needs to be taken in order not to lose specific sets of peptides (e.g. hydrophobic peptides binding to plastic).

### Generating alternative SWATH spectral libraries from the full spectral library

The current SWATH spectral library has been constructed for 64 variable windows selecting the six most intense fragment ions. However, a zebrafish SWATH spectral library with any other window configuration or transition selection can easily be performed based on the deposited full consensus spectral library using the spectrast2tsv.pv function as described previously^11^.

### Ensembl version of Peptide identifiers

We have used Ensembl version 91 from December 2017 (GRCz10) to map the peptides. As the Ensembl identifiers change with subsequent versions, the archived Ensembl database should be used when analyzing the data with this spectral library. In addition, we have included a function called (convert_protein_ids) in the R/Bioconductor package SWATH2stats 1.11.2^15^. This function supports the mapping of Ensembl peptide identifiers with the biomaRt package^35^ to Ensembl gene identifiers or other gene symbols.

### Estimation of false-discovery rate (FDR)

SWATH employs a peptide-centric data query strategy in which the false discovery rate (FDR) is estimated using so-called decoy peptides^8^. Naïve decoy counting cannot be applied, but post-analysis approaches exist to efficiently control the FDR with large SWATH spectral libraries^15,16^. In order for these approaches to work reliably, it is important that enough peptides are present in the samples to estimate the distribution of the discriminant score for the true targets. Hence, it is especially important to control this requirement when analyzing heavily fractionated samples with a large SWATH spectral library. Apart from that, the large number of peptides present in the spectral library should not have detrimental effects on the performance of querying the mass spectrometric data.

### Portability of the spectral library to other instruments

The described SWATH spectral library was generated on a TripleTOF instrument (Sciex TripleTOF 5600) with the described collision energy settings. To use the SWATH spectral library on a different instrument, the similarity of the fragmentation needs to be ensured which might include optimizing the collision energy. The similarity of the fragment spectra can be compared using our previously published tool^36^. In order to re-align the retention times, we recommend to spike so-called iRT peptides into the sample or use conserved peptides for retention time alignment^37,38^.

## Additional information

**How to cite this article**: Blattmann, P. *et al*. Generation of a zebrafish SWATH-MS spectral library to quantify 10,000 proteins. *Sci. Data*. 6:190011 https://doi.org/10.1038/sdata.2019.11 (2019).

**Publisher’s note**: Springer Nature remains neutral with regard to jurisdictional claims in published maps and institutional affiliations.

## Supplementary Material



## Figures and Tables

**Table 1:** Samples acquired for the zebrafish SWATH spectral library.

Tissue	Peptide fractionation	MS Samples	MS Injections
Brain	None	1	3
Brain	high-pH RP-HPLC	8	8
Eye	None	2	6
Eye	high-pH RP-HPLC	8	8
Heart	None	1	3
Heart	high-pH RP-HPLC	8	8
Intestine	None	1	3
Intestine	high-pH RP-HPLC	7	7
Liver	None	1	3
Liver	high-pH RP-HPLC	8	8
Muscle	None	3	3
Muscle	high-pH RP-HPLC	8	8
Ovary	None	1	3
Ovary	high-pH RP-HPLC	8	8
Spleen	None	1	3
Spleen	high-pH RP-HPLC	8	8
Testis	None	1	3
Testis	high-pH RP-HPLC	8	8
Total		83	101
Peptides from unfractionated peptide samples were injected in three technical replicates, fractionated peptide samples were only injected once. For the eye, two unfractionated peptide samples (the eye and the vitreous body) were processed.			

**Table 2:** Size of the zebrafish SWATH spectral library.

	Proteotypic	Proteotypic and Shared
**Proteins**	10,405	12,770
**Peptides**	104,185	114,912
**Precursors**	129,561	143,901
**Transitions**	777,366	863,406
Number of proteins, peptides, precursors, and transitions that pass a protein FDR of 1% are shown (see Methods). The number of proteins etc. supported by proteotypic as well as proteotypic and shared peptides are shown.		
